# Special Issue “COVID-19: Diagnostic Imaging and Beyond—Part II”

**DOI:** 10.3390/jcm11133786

**Published:** 2022-06-30

**Authors:** Chiara Giraudo, Isolde Martina Busch

**Affiliations:** 1 Department of Medicine—DIMED, University of Padova, 35128 Padova, Italy; 2 Section of Clinical Psychology, Department of Neuroscience, Biomedicine, and Movement Sciences, University of Verona, 37134 Verona, Italy; isoldemartina.busch@univr.it

More than two years have passed since the onset of the COVID-19 pandemic. During this difficult time, many challenges have been faced by the scientific community, aiming to assess and understand this new disease, and to treat affected patients safely, all while trying to contain the spread of the virus [1,2]. Especially the rapid development of effective vaccines represented an extraordinary achievement, leading to reduced morbidity and mortality worldwide [3,4]. Nevertheless, some obstacles still have to be overcome. Even if it has been reported that up to 67% of patients may develop fibrotic pulmonary changes, the role of prognostic factors as well as the impact of specific early treatment on this long-term consequence still have yet to be fully understood [5]. Moreover, it cannot be overlooked that the mere radiological detection of signs such as fibrotic bands may lead to an overestimation of this clinical entity [5]. Quantitative imaging, such as radiomics and artificial intelligence, which provided interesting results in terms of disease prognosis and pulmonary disease residues in the short and mid-term (e.g., three months), are expected to further contribute to predicting long-lasting fibrotic changes [6,7].

In addition to the various pulmonary signs and symptoms, we have learned that COVID-19 causes a systemic disease with a wide range of effects in adults and children, which is assessable by imaging [8,9,10,11]. Gastrointestinal symptoms with, for instance, signs of colitis at computed tomography (e.g., bowel wall thickening) [9,12], anosmia expressed as olfactory bulb anomalies at MR [10,13] or myocarditis-like features (e.g., myocardial edema and/or late gadolinium enhancement) at cardiac MR in children with multisystem inflammatory syndrome (MIS-C) due to COVID-19 [11,14] are just some examples of disease manifestation which can be radiologically identified. Such a wide spectrum of symptoms may occur and also persist in the so called Post-COVID syndrome or Long-COVID that, as recently demonstrated, may last over a year [15]. In fact, patients may experience enduring pulmonary, cardiovascular, endocrine, hematologic, gastrointestinal, neurological, and/or psychiatric signs [16]. For instance, neuroinflammatory mechanisms as well as the effects of microvascular injuries, detected at MR, are considered responsible for acute and persistent neurological symptoms [17,18].

Moreover, it is now well established from a variety of studies focusing on the psychiatric and psychological impact of COVID-19 that especially severe infections and long-standing disease can cause emotional distress for patients, family members, and healthcare providers [19,20]. Indeed, already in the early phases of the pandemic, studies showed that COVID-19 patients experience somatic symptoms, anxiety, depression, and sleeping problems [21]. Similar symptoms were reported by healthcare workers, including radiologists who have been, among other specialists from high-risk settings (e.g., emergency departments, infectious disease units, intensive care units) on the frontlines of the pandemic [19,22]. Considering this immense psychological burden, we should further investigate if and how healthcare institutions supported physicians during this difficult experience and which strategies have been implemented to prevent these effects in case of future infectious disease outbreaks. Today, healthcare workers are exposed to additional stress factors, represented by the backlog of clinical visits, surgical procedures, and imaging appointments [23]. In fact, in the beginning of the pandemic, policies curtailing for instance elective procedures and radiological examinations were applied but now an efficient response must be adopted regarding the management of this accumulation of cases [24,25,26]. Thus, it would be interesting to understand if tailored programs and strategies have been established in different countries to better forecast medical visits and diagnostic imaging demand, reduce the unnecessary deferral of care, and to recruit additional healthcare professionals.

At the same time, as an additional challenge, healthcare institutions are facing an exodus of healthcare professionals [27]. In fact, it seems that one in five healthcare workers has left their job since the beginning of the pandemic and, according to a recent survey [28], one third of nurses are willing to quit their job. The latter group reported high-stress work environments and burnout as the main reasons for quitting, followed by insufficient pay and missing benefits. Certainly, this complex phenomenon is not only related to COVID-19 but the pandemic added to pre-existing issues, such as understaffed units and heavy workload, then leading to a breaking point [28,29].

Despite a tremendous interest of the academic community in the underpinning mechanisms of COVID-19, several aspects regarding radiological imaging, as well as the clinical and psychosocial management of the disease and its long-lasting physical and psychological impact, still need to be examined and better understood. This Special Issue is a chance for radiologists and clinicians of different medical fields to increase our knowledge about the long-term consequences of COVID-19, the beneficial effects of vaccines and novel treatments, and the global impact of this pandemic on the well-being of patients and healthcare providers as well as the overall healthcare system. Review articles about the current evidence as well as original articles presenting novel findings are very welcome and will certainly improve our clinical practice. Research addressing psychosocial aspects will represent a source of growth for healthcare professionals.

Our gratitude goes out to all researchers contributing to this Special Issue and to the readers who will then have the chance to bridge the gap between research-based knowledge and clinical practice.

## Data Availability

Not applicable.
